# Detection of Ampicillin-Resistant *E. coli* Using Novel Nanoprobe-Combined Fluorescence In Situ Hybridization

**DOI:** 10.3390/nano9050750

**Published:** 2019-05-16

**Authors:** Wang Sik Lee, Soohyun Lee, Taejoon Kang, Choong-Min Ryu, Jinyoung Jeong

**Affiliations:** 1 Environmental Disease Research Center, Korea Research Institute of Bioscience Biotechnology (KRIBB), Daejeon 34141, Korea; wang3026@kribb.re.kr; 2 Department of Nanobiotechnology, KRIBB School of Biotechnology, UST, Daejeon 34113, Korea; 3 Infectious Disease Research Center, KRIBB, Daejeon 34141, Korea; ysh76@kribb.re.kr; 4 Bionanotechnology Research Center, KRIBB, Daejeon 34141, Korea; 5 Department of Biosystems and Bioengineering, KRIBB School of Biotechnology, UST, Daejeon 34113, Korea

**Keywords:** antibiotic-resistance, ampicillin, bacteria, fluorescence in situ hybridization, nanoprobe

## Abstract

Antibiotic-resistant bacteria present a global threat because the infections they cause are difficult to treat. Therefore, it is highly important to develop advanced methods for the identification of antibiotic resistance gene in the virulent bacteria. Here, we report the development of novel nanoprobes for fluorescence in situ hybridization (FISH) and the application of the nanoprobe to the detection of ampicillin-resistant *Escherichia coli*. The nanoprobe for FISH was synthesized by the modified sol–gel chemistry and the synthesized nanoprobe provided strong fluorescent signals and pH stability even under natural light condition. For the double-identification of bacteria species and ampicillin-resistance with a single probe in situ, the nanoprobes were conjugated to the two kinds of biotinylated probe DNAs; one for *E. coli*-species specific gene and the other for a drug-resistant gene. By using the nanoprobe-DNA conjugants, we successfully detected the ampicillin-resistant *E. coli* through the FISH technique. This result suggests the new insight into light stable FISH application of the nanoprobe for a pathogenic antibiotic-resistance bacterium.

## 1. Introduction

Antibiotic-resistant bacteria have been a global issue threatening public health because they are increasingly difficult to treat, leading to higher medical costs, prolonged hospital stays, and increased mortality [1]. Each year in the U.S., at least 2 million people get an antibiotic-resistant infection and at least 23,000 people die, resulting in a 55–70 billion dollar per year economic impact [2]. Antibiotic resistance in bacteria can be induced both from mutations arising in chromosomal genes and acquisition of mobile genetic elements [3]. Therefore, it is critical to identify the pathogen species and drug-resistant gene accurately in a timely manner for appropriate treatment of antibiotic-resistant bacteria infected patients. For the diagnostics of antibiotic-resistant bacteria, the conventional culture-based plating assay has been widely used. However, this method requires several days to confirm the growth of the targeted bacterial colony [4]. In comparison, a molecular-based detection method such as polymerase chain reaction (PCR) requires relatively less time than the culture-based plating assay, but still cannot fully avoid separation and bacterial pre-enrichment [5]. In addition, matrix assisted laser desorption ionization time-of-flight spectrometry has attracted interest for the rapid identification of pathogens by profiling bacterial proteins from the whole cells [6]. Moreover, endogenous H_2_S evolution was recently developed for drug-resistant bacteria via in situ hybridization [7]. Since the diagnostic tests are crucial to the management of infectious diseases and combatting the rise in antibiotic resistance, it is urgent to develop the advanced diagnostic methods, which would need to be simple, rapid, accurate, and low cost, for the detection and profiling of antibiotic resistance genes or microorganisms.

Fluorescence in situ hybridization (FISH) is a technique for the identification and analysis of diverse organisms such as bacteria and animal cells, based on the hybridization of fluorescently labeled oligonucleotide probe to complementary target sequences from organisms using epifluorescence or confocal laser scanning microscopy [8,9]. Since FISH has several advantages including simplicity, selectivity, rapidness, and non-cultivation, it has been widely used to detect ribosomal RNA (rRNA) of pathogenic bacteria in clinical communities [10]. Moreover, FISH also has been used to identify the antimicrobial drug-resistant bacteria (e.g., macrolide-resistant *Helicobacter pylori* and chloramphenicol-resistant *Bacillus cereus*) [11,12]. However, there are considerable factors for the optimization of target DNA hybridization during FISH such as hybridization time and temperature [13]. In addition, weak and unstable fluorescent signals due to quenching caused by natural and artificial light remain as the limitation for the detection of a single microbe using fluorescence microscopy. Recently, advanced FISH techniques—such as catalyzed reporter deposition-FISH, nanogold-based FISH, and peptide nucleic acid based-FISH—have been developed to overcome these drawbacks [14,15,16,17]. While these methods enhanced the fluorescent signals and improved the detection capability of FISH, there are still requirements to develop a novel FISH probe which provides high stability, strong fluorescence, and simple modification. Additionally, the double-identification of bacteria species gene and antibiotic resistance gene with a probe rarely reported.

Herein, we developed a novel fluorescent nanoparticle-based probe (nanoprobe) for FISH technique and successfully applied the nanoprobe for the detection of antibiotic-resistant bacteria. The stable nanoprobe was prepared by the modified sol–gel chemistry and consisted of fluorescent dye-loaded poly(d,l-lactide-co-glycolide) (PLGA) and silica nanoparticles (NPs) [18,19]. The densely loaded fluorescent dyes in a nanoprobe provided strong fluorescent signals and the silica NPs enabled us the simple modification of ssDNA. We characterized the nanoprobe by measuring transmission electron microscopy (TEM), Fourier transform infrared spectroscopy (FTIR), and fluorescence. The developed nanoprobe showed the significantly improved pH stability, which are highly advantageous for FISH. For the identification of ampicillin-resistant *Escherichia coli*, the nanoprobe was functionalized with two kinds of biotinylated single stranded DNAs (ssDNAs) which can conjugate to *E. coli*-specific gene and ampicillin-resistance bla gene that encodes beta-lactamase conferring beta-lactame (e.g., ampicillin) degrading enzyme, respectively. Finally, we successfully detected ampicillin-resistant *E. coli* using a nanoprobe-ssDNA. To the best of our knowledge, this is the first report of nanoprobe-combined FISH method for the detection of drug-resistant bacteria. We anticipate that the stable nanoprobe can broaden the capability of FISH technique and further be used for the various kinds of biological applications.

## 2. Materials and Methods

### 2.1. Materials

Tetraethyl orthosilicate (TEOS), (3-amiopropyl) trimethoxysilane (APTMS), cyclohexane anhydride, L-arginine, fluorescein isothiocyanate (FITC), rhodamine-B-isothiocyanate (RITC), acetone, ethanol, and dimethyl sulfoxide (DMSO) were purchased from Sigma-Aldrich (St. Louis, MO, USA). PLGA was purchased from Boehringer Ingelheim (Ingelheim am Rhein, Germany). Streptavidin (SA) and SA-Alexa647, phosphate buffered saline (PBS) were purchased from Invitrogen (Carlsbad, CA, USA). N-hydroxylsuccinimide (NHS)-polyethylene glycol (PEG)-biotin (NHS-PEG12-biotin), methyl-PEG-NHS were ordered from Thermo (Waltham, MA, USA). The ss-DNA probe (biotin-AAAAAAAAAAGCWGCCWCCCGTAGGWGT) and the complementary ss-DNA-Cy3 were synthesized by Bioneer, Inc. (Daejeon, Korea).

### 2.2. Synthesis of Nanoprobe

The fluorescent PLGA-silica NPs were synthesized by modified water-in-oil (W/O) method [20,21]. Briefly, the PLGA-FITC was prepared by mixing 200 µL of FITC (2 mg in acetone), 1.8 mL of PLGA (40 mg in acetone), and 2 mL of distilled water under stirring for 2 h at room temperature. Then 444 µL of 10 mM APTMS (final concentration of 1 mM) was added to the PLGA-FITC and reacted for 3 h. Next, the mixture including 1 mL of cyclohexane, 7 mL of distilled water, 100 µL of TEOS, and 10 mg of L-arginine was added to the PLGA-FITC suspension and stirred for overnight at 70 °C. Finally, 70 µL of APTMS (1 M) was added and mixed for 3 h at room temperature. After the reaction and washing with distilled water three times, the product was dispersed in PBS (pH 7.4). The R-nanoprobe was synthesized through the same method by using RITC instead of FITC. 

### 2.3. Preparation of Nanoprobe-DNA

The target DNA-conjugated nanoprobes was prepared by amine reactive crosslinking [22,23]. The amine-modified nanoprobe (1 mg/mL) was reacted with 100 µL of NHS-PEG12-Biotin (1 mM) in phosphate buffer for 1 h at room temperature. The sample was centrifuged and washed three times with phosphate buffer to remove the unbound biotin. Then, 100 µL of NHS-PEG (1 mM) was added to the nanoprobe-biotin solution as a blocking reagent and reacted for 30 min. The solution was centrifuged to remove the free NHS-PEG. Next, 50 µL of SA (1 mg/mL) was mixed with the nanoprobe-biotin for 1 h and rinsed by centrifugation. Finally, 100 µL of ss-DNA probe (1 uM) was reacted with the nanoprobe-biotin-SA for 1 h. The resultant nanoprobe-DNA was washed by centrifugation and dispersed in PBS (pH 7.4).

### 2.4. Design of Oligonucleotide Probes

Oligonucleotide probes were designed with Integrated DNA Technologies software (IDT, Coralville, IA, USA), the universal bacteria detection probe of rrnB sequences was obtained from conserved 16S rRNA with a sufficiently high cellular ribosome content of bacteria. The target antibiotic resistance probe of bla sequences was obtained from bla gene, encoded beta-lactamase in the plasmid pUC19 (accession no. M77789) [24]. The oligonucleotide probes were purchased from Genotech (Genotech Co., Daejeon, Korea).

### 2.5. Bacterial Strains and Culture Conditions

The *E. coli* strains were grown in Luria–Bertani (LB) broth (Difco, Franklin Lakes, NJ, USA, Cat. no. 244620) or LB broth with 100 µg/mL ampicillin (Sigma-Aldrich, St. Louis, MO, USA, Cat. no. A0166) until exponential phase at 37 °C and 200 rpm. The *E. coli* DH5α and plasmid pUC19 were purchased from Invitrogen (Carlsbad, CA, USA, Cat. no. 18258-012).

### 2.6. Cell Fixation

The bacterial cell fixation was performed by modified fixation method [25]. Cultured bacterial cells were harvested by centrifugation at 12,000 rpm for 1 min and discarded the supernatant. Cell pellets were washed with PBS (Biosesang Inc. Co., Seongnam, Korea, Cat. No. P2004) three times. For the permeabilization of the cell, ice-cold 4% paraformaldehyde in PBS was applied for 3–12 h at 4 °C. For the fixation of bacterial cells, the permeabilized cells were resuspended with ice-cold 50% EtOH. Next, 10 μL of fixed cell suspension was dropped on the gelatin coated-slide glass and dried under air flow. The slide glass was soaked in the solution of 50% EtOH for 3 min, 80% EtOH for 3 min, and 95% EtOH for 3 min. Finally, the slide glass was air dried.

### 2.7. Hybridization of Nanoprobe-DNA

The detection of antimicrobial bacteria using nanoprobe-DNA was conducted by modified common method [26]. The pre-warmed 200 μL of hybridization buffer (0.9 M NaCl, 20 mM Tris-Cl, 0.01% sodium dodecyl sulfate (SDS), and formamide, pH 8.0) was mixed with 10 pmol of the nanoprobe-DNA and carefully applied to the fixed cell on the slide glass. The binding stringency of the applied nanoprobe-DNA against target genes in *E. coli* was adjusted by varying the formamide concentration from 0 to 80%. The applied slide glass was covered with cover glass and sealed with Cytobond (SciGene, Sunnyvale, CA, USA, Cat. no. 2020-00-1) for avoiding dehydration. After hybridization for 4 h at 46 °C in a Denaturation and Hybridization System (TDH-500, Allsheng Ins. Co., Hangzhou, China), the cover glass was removed and the slide glass was rinsed with washing buffer (5 mM ethylenediaminetetraacetic acid, 20 mM Tris-Cl, 0.01% SDS, and NaCl solution, pH 8.0). To remove non-specific bindings, the stringency was adjusted by varying the NaCl solution from 0 to 80%, depending on the used formamide concentrations, for 10 min at 48 °C and rinsed with ice-cold water.

### 2.8. Instrumentation

FTIR measurements were carried out with ALPHA-T (BRUKER Inc. Billerica, MA, USA). TEM images were observed using a JEM-2100F, 200 kV (JEOL LTD., Tokyo, Japan). Dynamic light scattering (DLS) and zeta-potential were conducted on an ELS-Z (Otsuka Electronic CO., LTD., Tokyo, Japan). Fluorescence spectra and intensity values were recorded on the FS-2 (SINCO CO., LTD., Daejeon, Korea) and spectraMax M2e. (Molecular Devices, LLC., San Jose, CA, USA). An upright fluorescence microscope (Nikon, Eclipse Ni, Tokyo, Japan) and digital camera system DS-Ri1 (Nikon, DS-Ri1, Tokyo, Japan) were used for the visualization of the bacteria for FISH. Fluorescence filters are longpass: green (excitation filter 460–500, dichroic mirror 505, and barrier filters 510–560), red (excitation filter 540/25, dichroic mirror 565, and barrier filters 605/55). 

## 3. Results and Discussion

**Figure 1:**
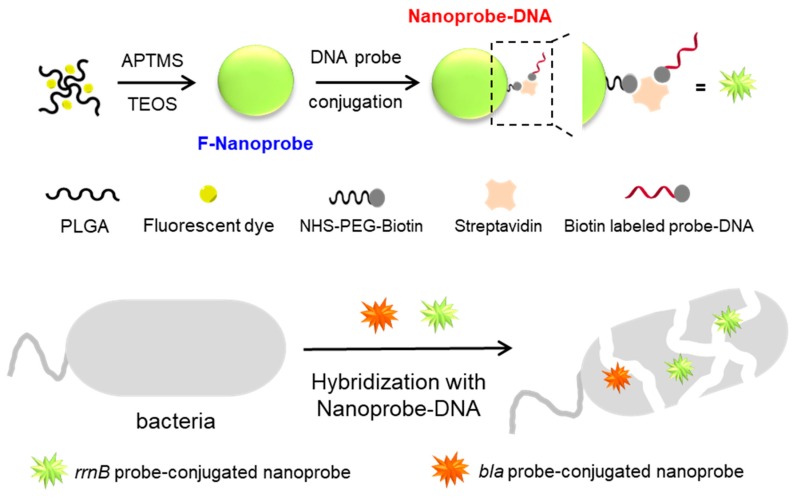
Schematic illustration of preparation of stable nanoprobe-DNA for FISH.

Figure 1 shows the schematic illustration of the preparation of stable nanoprobe-ssDNA for FISH. The modified W/O micro-emulsion method was employed to synthesize the nanoprobe. First, PLGA and fluorescent dyes (FITC or RITC) were mixed to form PLGA-Dye via hydrophobic interaction. Since the fluorescent dyes are limitedly soluble and decompose in water, PLGA was used for the enrichment and increase of the stability of dyes. Then, PLGA-Dye was conjugated with APTMS via thiourea linkage between the isothiocyanate (−N=C=S) group of dye and the primary amine (−NH_2_) group of APTMS. Next, silanization was accomplished by the reaction with TEOS in a mixture of cyclohexane and aqueous solution containing L-arginine, acting as a surfactant to form a micro-emulsion. Lastly, the stable nanoprobe was collected by centrifugation. In order to conjugate the nanoprobe with ssDNA, we modified the nanoprobe with APTMS. The resultant amino-modified nanoprobe was then reacted with NHS-PEG-Biotin for 1 h at room temperature. After the addition of blocking reagent (NHS-PEG), the nanoprobe was reacted with SA and biotinylated probe DNA sequentially. The final nanoprobe-ssDNA was washed by centrifugation and dispersed in PBS (pH 7.4).

**Figure 2:**
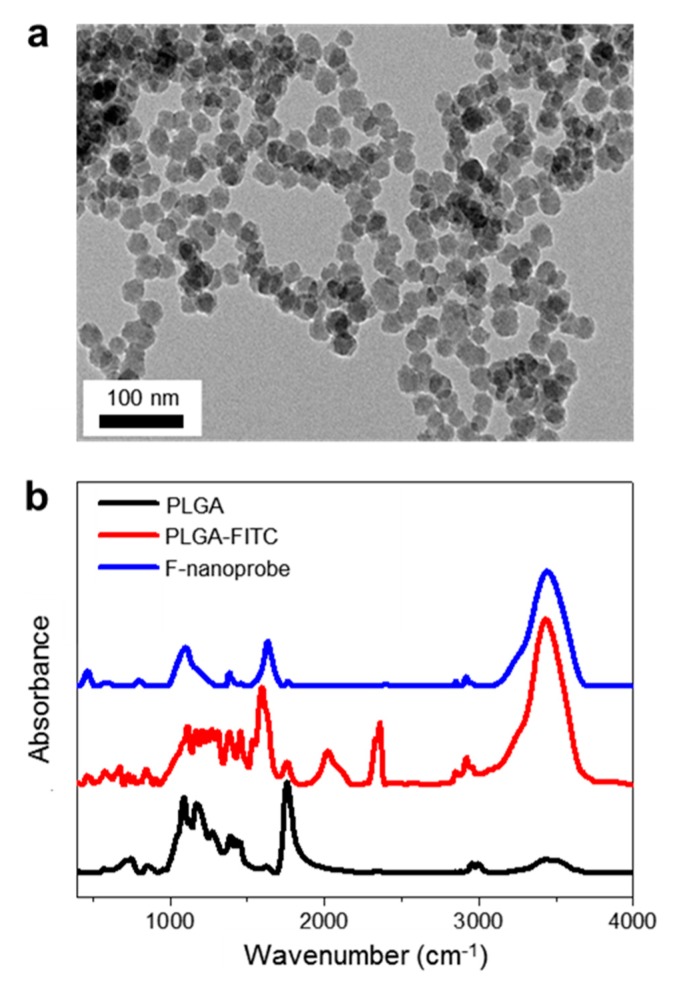
(**a**) TEM image of F-nanoprobes. (**b**) FTIR spectra of PLGA (black), PLGA-FITC (red), and F-nanoprobe (blue).

Figure 2a is a TEM image of the nanoprobes including FITC (F-nanoprobes). The nanoprobes were highly homogeneous and have diameters of 23.65 ± 2.19 nm. We also measured the hydrodynamic diameters of PLGA-FITC and F-nanoprobe, respectively. Average hydrodynamic diameter of PLGA-FITC was obtained as 71.96 ± 0.35 nm and that of F-nanoprobes was 35.27 ± 3.06 nm, suggesting that the nanoprobe has densely loaded fluorescent dyes. Figure 2b is FTIR spectra of PLGA, PLGA-FITC, and F-nanoprobe. C–O and C=O stretching vibration peaks at 1634 cm^−1^ and 1764 cm^−1^ were consistently observed in both spectra of PLGA and PLGA-FITC. The isothiocyanate peak at 2125 cm^−1^ was only observed in the spectrum of PLGA-FITC, indicating the successful encapsulation of FITC in PLGA. Moreover, the characteristic peaks of the silica NPs were shown at 811 cm^−1^ (Si–OH) and 1110 cm^−1^ (Si–O–Si) in the spectrum of F-nanoprobe. The TEM and FTIR analysis results prove the successful synthesis of a nanoprobe. 

**Figure 3:**
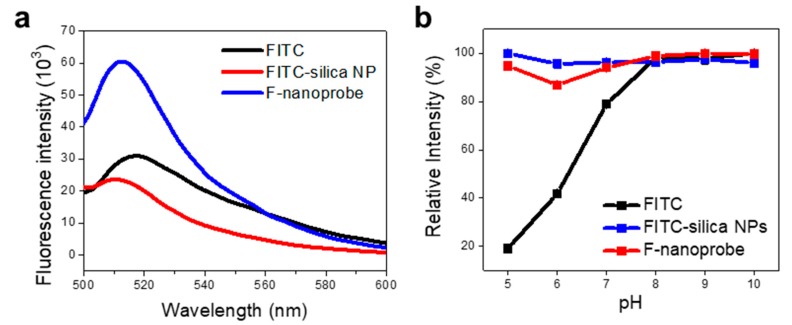
(**a**) Fluorescence spectra of FITC (black), F-nanoprobe (blue) and FITC-silica NPs prepared without PLGA (red). (**b**) Plots of relative fluorescence intensity versus pH for FITC (black), F-nanoprobe (blue), and FITC-silica NPs without PLGA (red).

Next, we obtained the fluorescence spectrum of F-nanoprobe as shown in Figure 3a (blue spectrum). Strong emission band at 515 nm was clearly observed from F-nanoprobe, indicating that this nanoprobe is suitable for FISH. Additionally, we prepared the FITC-silica NPs without PLGA as described in previous literature and measured the fluorescence spectrum of the FITC-silica NPs without PLGA (red spectrum in Figure 3a) for comparison. As shown in Figure 3a, the FITC-silica NPs without PLGA provided relatively weak band than F-nanoprobe. This represents that PLGA can lead the accumulation of fluorescent dyes efficiently, thus enhancing the fluorescent signals of nanoprobe. Considering that the weak fluorescent signals remain as the limitation of FISH technique for the detection of a single microbe, this strongly fluorescent nanoprobe can be highly attractive for FISH. We also examined the pH-stability of the F-nanoprobe, FITC-silica NPs without PLGA and FITC. For the application of nanoprobe to FISH technique, the strong fluorescent signals of nanoprobe should be preserved without quenching. Figure 3b shows the plots of relative fluorescence intensity of three samples versus pH from 5 to 10. The fluorescent signals of F-nanoprobe were well maintained in a wide range of pH. The FITC-silica NPs without PLGA also provided the stable signals through the whole pH. On the other side, the fluorescence intensity of FITC remarkably decreases from pH 8 to pH 5. This suggests that silica NP encapsulation of dyes can improve the pH-stability of nanoprobe significantly. This pH-independent nanoprobe can be advantageous for acting as a FISH probe which works in internal bacteria after permeabilization [15].

**Figure 4:**
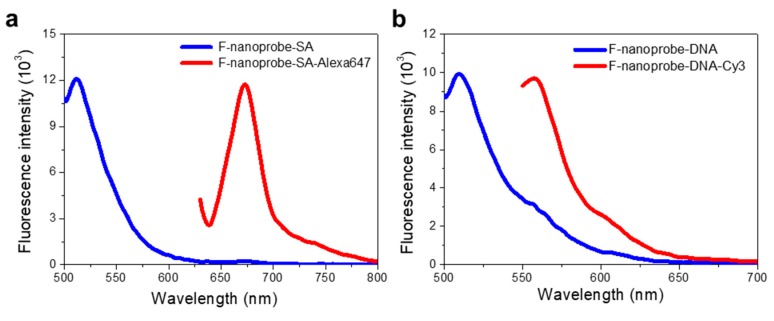
(**a**) Fluorescence spectra of F-nanoprobe-SA (blue) and F-nanoprobe-SA-Alex647 (red). (**b**) Fluorescence spectra of F-nanoprobe-DNA (blue) and F-nanoprobe-DNA-Cy3 (red).

For the application of nanoprobe to FISH technique, the nanoprobe should be functionalized with ssDNA that can bind to complementary target sequence in an organism. In this experiment, we employed the ampicillin-resistant *E. coli* as a model antibiotic-resistant bacteria and designed two kinds of probe DNAs for the detection of ampicillin-resistant *E. coli*. One probe DNA is rrnB gene for bacteria species specific target and another probe DNA is bla gene for antibiotic resistance-specific target. Table 1 shows the sequences of the probe DNAs. rrnB gene is a universal bacteria sequence from conserved 16S rRNA and bla gene is encoded beta-lactamase in plasmid pUC19 [24]. As depicted in Figure 1, the probe DNAs can be conjugated to the nanoprobes via sequential treatments of APTMS, NHS-PEG-biotin, SA, and biotinylated probe DNAs. Firstly, we treated the F-nanoprobe with APTMS and measured the zeta potential of the nanoprobe before and after the treatment. The F-nanoprobe exhibited the highly negative-charged value of −30.54 mV. After the reaction of F-nanoprobe with APTMS, the nanoprobe became less negatively charged (−16.75 mV). This indicates that amine functionalization was successfully accomplished on the F-nanoprobe. Next, the amine functionalized nanoprobe was conjugated with NHS-PEG-biotin routinely. The resultant nanoprobe-biotin was then reacted with SA, producing the nanoprobe-SA. Figure 4a displays the fluorescence spectrum of the F-nanoprobe-SA (blue spectrum). As same as the bare F-nanoprobe, strong emission band at 515 nm was observed from F-nanoprobe-SA. To verify the conjugation of SA on the F-nanoprobe clearly, we conjugated Alexa647-labeled SA to the F-nanoprobe and obtained the fluorescence spectrum of nanoprobe-SA-Alexa647 (red spectrum in Figure 4a). The strong emission band at 660 nm reveals that Alexa647-labeled SA was successfully conjugated to the nanoprobe. Since the biotin and SA interaction has a high binding affinity (Ka ~1015 M^−1^) and SA can bind up to four molecules of biotin, the final nanoprobe-DNA could be constructed by simply mixing the nanoprobe-SA and the biotinylated probe DNA (biotin-AAAAAAAAAAGCWGCCWCCCGTAGGWGT) [27]. We also obtained the fluorescence spectrum of the F-nanoprobe-DNA with maximum at 515 nm (blue spectrum in Figure 4b). To investigate the capability of the F-nanoprobe-DNA for FISH, the F-nanoprobe-DNA was hybridized to the Cy3-labeled complementary DNA. Figure 4b shows the fluorescence spectrum of F-nanoprobe-DNA after hybridization of Cy3-labeled DNA (red spectrum). The strong band at 560 nm suggests that the nanoprobe-DNA successfully hybridized to the Cy3-labeled complementary DNA, verifying the capability of nanoprobe-DNA for FISH. The number of DNA conjugated on a single nanoprobe was calculated to be 17.3 based on the standard curve of DNA by measuring absorbance at 258 nm. This result prompted us to employ the novel nanoprobe-DNA for the detection of antibiotic-resistant bacteria through FISH technique.

**Figure 5:**
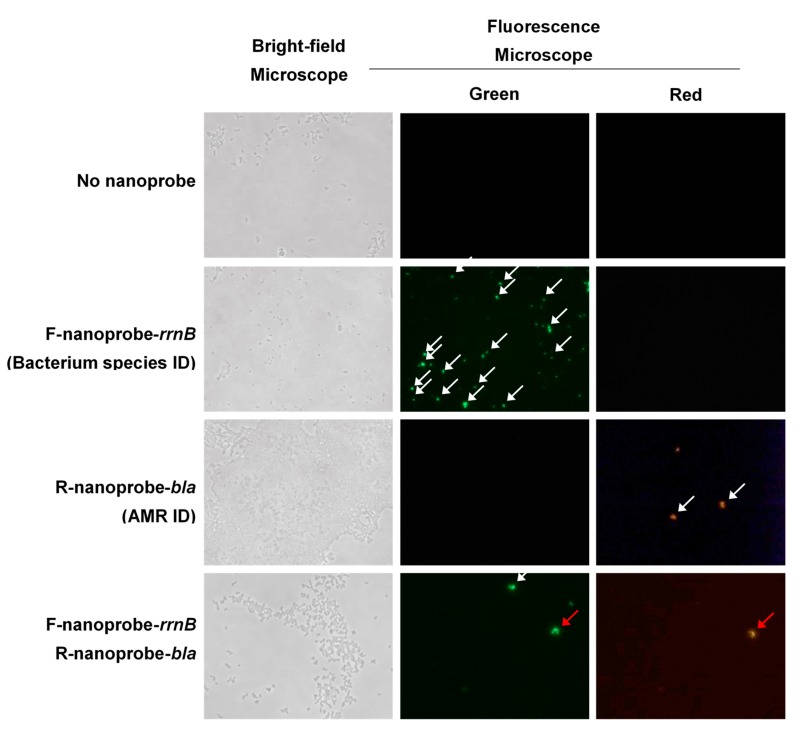
Bright-field and fluorescence images of *E. coli* for the identification of bacterium species and antimicrobial resistance (ampicillin-resistant) *E. coli* using the nanoprobe-combined FISH method. F-nanoprobe-rrnB recognizes 16s rDNA sequence (Bacterium species ID) of *E. coli* and provides green fluorescence signals in the second row. R-nanoprobe-bla recognizes beta-lactamase sequence of ampicillin-resistant *E. coli* and provides red fluorescence signals the third row. The fourth row was applied both F-nanoprobe-rrnB and R-nanoprobe-bla. The first row indicates the background images under bright-field and fluorescence microscope. The white arrows marked *E. coli* cells containing single gene rrnB or bla under fluorescence microscope. The red arrow indicates an *E. coli* cell containing both rrnB and bla genes.

Finally, we examined the FISH-based identification of bacterium species and ampicillin-resistant (antibiotic resistance, AMR) *E. coli* by using the nanoprobe. For the discrimination of rrnB and bla genes in *E. coli*, F-nanoprobe and R-nanoprobe were prepared, respectively. The F-nanoprobe-rrnB for bacterium species identification (bacterium specific ID) can detect 16s rDNA sequence of *E. coli* and provide green fluorescent signals (the second row in Figure 5). The R-nanoprobe-bla for antimicrobial resistance identification (AMR ID) can detect beta-lactamase sequence of ampicillin-resistant *E. coli* and provide red fluorescent signals (the third row in Figure 5). Figure 5 shows the bright-field and fluorescence images of *E. coli* for the identification of species and ampicillin-resistant *E. coli* using the nanoprobe-combined FISH method. Under bright-field monitoring, *E. coli* was observed clearly as shown in the left column of Figure 5. Without nanoprobes, no fluorescent images through green and red fluorescence field were obtained (first row-middle and right column in Figure 5) indicating that no background for FISH was detected under microscopes. In the presence of F-nanoprobe-rrnB, green fluorescent signal was clearly observed (second row-middle column of Figure 5) but red fluorescent signals were negligible (second row-right column of Figure 5). In the presence of R-nanoprobe-bla, a red fluorescent signal was shown (third row-middle column of Figure 5) but there were no green fluorescent signals (third line-right column of Figure 5). The green and red fluorescence signals (white arrows) indicate *E. coli* cells that contained rrnB for bacterium specific ID and bla AMR ID, respectively. Lastly, we obtained the fluorescent images of *E. coli* in the presence of both F-nanoprobe-rrnB and R-nanoprobe-bla. The red arrow marked *E. coli* in fourth column of Figure 5 provides green and red fluorescent signals simultaneously in the same *E. coli* cell, indicating that this novel nanoprobe-combined FISH-method enable detect bacterium species and AMR ID at the same time under in situ condition. Furthermore, it is noteworthy that the nanoprobes are highly stable even under the natural light conditions, while conventional fluorescent dyes are fade out shortly under the same conditions. The white arrow in the fourth row-middle column of Figure 5 indicates an *E. coli* cell that was detected only rrnB gene without bla gene (Figure 5). We anticipate that the present nanoprobe-based FISH technique can be used for the detection pathogenic bacteria species containing specific drug-resistant genes, preventing the infection of antibiotic-resistant bacteria.

## 4. Conclusions

We developed a novel nanoprobe for FISH method by merging the fluorescent dye-loaded PLGA and silica NPs. The nanoprobe exhibited the strong fluorescent signals, high pH and light stability, and easy ssDNA functionalization, making the nanoprobe advantageous for FISH. For the application of the nanoprobe to the detection of both pathogenic bacteria species and antibiotic-resistance gene, we prepared F-nanoprobe containing rrnB gene-specific DNA and R-nanoprobe containing bla gene-specific DNA, respectively. By using the nanoprobe-combined FISH approach, the ampicillin-resistance *E. coli* could be identified by microscopic observation. We anticipate that the nanoprobe-based FISH method will be employed for the detection of various kinds of pathogenic bacteria, and diagnosis of the emergence of the infectious drug-resistance bacteria.

## Figures and Tables

**Table 1:** Oligonucleotide sequences used in the experiments

Gene Names	Sequences (5′-3′) *^a^*	Target
rrnB	GCWGCCWCCCGTAGGWGT	Eubacteria
bla	AGTGGTCCTGCAACTTTATCCCTCCGATCGTTGTCAGAAGTAAG	Ampicillin-resistance

*^a^* The oligonucleotides were modified by biotin-(A)_10_ at 5′-termini.
